# High-Sensitivity Terahertz Time-Domain Spectroscopic Characterization of the Thermal Evolution of Hydrated Copper Sulfate

**DOI:** 10.3390/molecules31081342

**Published:** 2026-04-19

**Authors:** Yuqiu Jiao, Xinyu Li, Yuqi Zhang, Qingying Xie, Yuhong Xia

**Affiliations:** College of Science, College of New Energy and Materials, China University of Petroleum (Beijing), Beijing 102249, China; lixy1359@gmail.com (X.L.); 18951235875@163.com (Y.Z.); xieqingyingcup@163.com (Q.X.)

**Keywords:** terahertz time-domain spectroscopy, CuSO_4_·5H_2_O, thermal decomposition, thermal evolution

## Abstract

To elucidate the influence of water on terahertz (THz) spectral responses, terahertz time-domain spectroscopy (THz-TDS) was employed to monitor the thermal decomposition of copper(II) sulfate pentahydrate in this study. Continuous dehydration of the hydrate induces pronounced variations in the THz signal. At the initial stage of thermal decomposition, these changes primarily originate from the evolving state and amount of water confined within the CuSO_4_·5H_2_O lattice. After detaching from the crystalline framework, the released water molecules do not evaporate immediately; instead, they transiently reside near the copper sulfate as free water. When the temperature reaches approximately 60 °C, a dynamic equilibrium is established between crystalline water and free water. The THz spectral data reveal that the sample exhibits its strongest THz absorption at this temperature. Consequently, the THz signal during decomposition displays a characteristic trend: an initial decrease followed by an enhancement. These findings demonstrate that THz-TDS represents a promising approach for probing the state and content of water, thereby contributing to the development of a powerful analytical tool for fundamental studies in mineralogy.

## 1. Introduction

Water is ubiquitous in nature and is readily incorporated into mineral structures through hydration processes, a phenomenon common to a wide range of minerals. The amount and structural forms of water hosted within minerals are highly diverse and often remarkably complex [1]. Over long periods of sedimentation and geological evolution, both the water content and the state of water in minerals can undergo significant modification [2]. Temperature is one of the most critical factors governing the abundance and configuration of water in mineral systems [3,4]. Water in minerals can generally be categorized according to its physical state and its relationship to the crystalline framework. The most frequently encountered types include adsorbed water, crystalline water, and structural water [5]. Adsorbed water is typically released at temperatures of approximately 100–200 °C. As the temperature increases further, crystalline water and subsequently structural water are liberated sequentially from the mineral matrix [6,7]. The removal of crystalline and structural water induces pronounced changes in the lattice structure, as structural water occupies fixed positions, quantities, and ratios within the crystal framework. Consequently, the physicochemical properties of minerals are highly sensitive to the presence and configuration of crystalline water [8]. Therefore, qualitative and quantitative characterization of water in minerals is of considerable significance for advancing fundamental mineralogical research.

The study of water in minerals has been approached through various analytical techniques, including thermogravimetric analysis (TGA), infrared spectroscopy, and nuclear magnetic resonance [9]. TGA provides quantitative information on water content and dehydration temperatures, while vibrational spectroscopies offer insights into the bonding states of water molecules [10]. However, these methods often face limitations in detecting subtle changes in water states or require complex sample preparation. For instance, infrared spectroscopy may struggle with strongly absorbing samples, and TGA alone cannot distinguish between different structural arrangements of water molecules.

In recent years, terahertz time-domain spectroscopy (THz-TDS) has attracted widespread attention in mineralogical research due to its ability to capture rich information on molecular structure and intermolecular interactions [11,12,13,14,15,16,17]. Polar molecules, such as water, exhibit strong absorption in the terahertz frequency range, enabling THz-TDS to serve as an effective probe for identifying both the state and quantity of water in materials [18,19]. Recent advances in THz-TDS have enabled its application in diverse fields such as pharmaceuticals, explosives detection, and cultural heritage conservation, where it has been increasingly used for non-destructive analysis of layered structures and degradation products [20,21,22,23]. In mineralogy, THz-TDS has been used to study hydrated minerals including gypsum, zeolites, and clay minerals [24]. The strong interaction between THz waves and hydrogen-bond networks makes this technique particularly suitable for probing water dynamics in crystalline hydrates [25]. For example, studies on ice [26] and aqueous solutions have demonstrated the sensitivity of THz radiation to the collective motions of water molecules and their hydrogen-bonding environments. Furthermore, THz-TDS has been successfully applied to monitor thermal decomposition processes in oil shale [26,27] and to probe the evolution of kerogen [28], highlighting the potential of THz-TDS for studying temperature-induced structural changes in complex mineral systems.

The crystalline water in copper(II) sulfate pentahydrate (CuSO_4_·5H_2_O) is highly sensitive to temperature, and the five water molecules are bonded to the CuSO_4_ units in distinct coordination environments. Using CuSO_4_·5H_2_O as a model system helps minimize compositional complexity and isolate the spectral contributions associated with water. The dehydration mechanism of CuSO_4_·5H_2_O has been extensively studied using conventional methods. Early crystallographic studies [29,30] established the crystal structures of the pentahydrate, trihydrate, and anhydrous forms, revealing the distinct coordination environments of the water molecules. Thermogravimetric analyses [10,31] demonstrated that dehydration proceeds in a stepwise manner, with the loss of two water molecules occurring at relatively low temperatures (below 100 °C), followed by the removal of another two water molecules between 100 and 200 °C, and finally the release of the last water molecule above 200 °C. Spectroscopic techniques such as infrared spectroscopy [10] have provided insights into the molecular-level changes during thermal treatment, showing progressive weakening of O-H stretching bands corresponding to water loss. However, these techniques have limitations in detecting intermediate states where released water molecules may temporarily exist as free water before evaporation, a phenomenon that THz-TDS is uniquely positioned to investigate due to its sensitivity to both bound and free water [31,32].

A previous THz-TDS study by Ma et al. [33] investigated the dehydration of CuSO_4_·5H_2_O and successfully distinguished different hydration states based on absorption coefficient changes. However, the detailed evolution of THz time-domain signals during the early dehydration stage, particularly the dynamic interplay between lattice-bound water and transient free water, has not been fully explored. In this work, we extend these findings by systematically analyzing the THz peak amplitude over a broad temperature range, revealing a characteristic decrease followed by an increase that indicates the establishment of a dynamic equilibrium between crystalline water and free water near 60 °C, a phenomenon that has not been reported.

In this study, THz-TDS was employed to investigate the thermal decomposition and dehydration behavior of copper(II) sulfate pentahydrate. The terahertz absorption characteristics of the decomposition products at different temperatures were systematically examined, revealing a strong dependence on the state and content of water. The results demonstrate the considerable potential of THz-TDS as a novel analytical method for probing water in minerals, offering valuable insights for advancing fundamental mineralogical research.

## 2. Results and Discussion

### 2.1. Terahertz Time-Domain Spectroscopic Analysis of the Thermal Decomposition of Crystalline Water

Because the crystalline water in copper(II) sulfate pentahydrate is highly sensitive to temperature, and the five water molecules are coordinated to the CuSO_4_ units in distinct manners, using CuSO_4_·5H_2_O as the study system minimizes the influence of compositional complexity on the experimental results. Under ambient conditions, CuSO_4_·5H_2_O is relatively stable; however, when the environmental humidity decreases, it gradually loses its crystalline water and eventually transforms into anhydrous copper(II) sulfate. Upon further heating, the anhydrous sulfate undergoes thermal decomposition at approximately 650 °C, producing copper oxide, sulfur dioxide, and oxygen.

**Figure 1:**
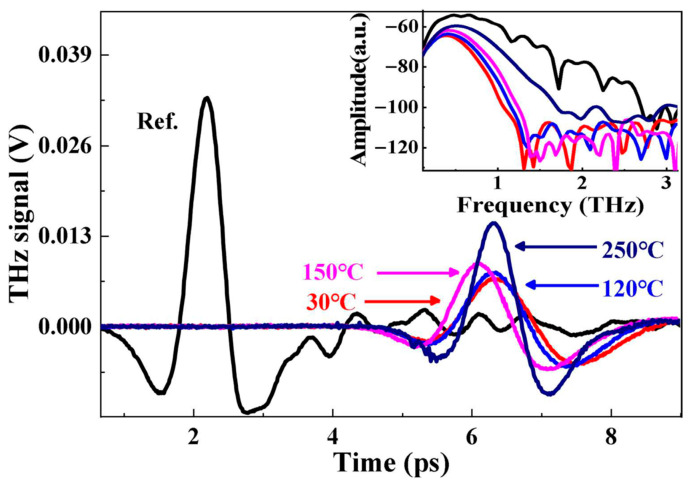
Terahertz time-domain spectroscopy of copper sulfate pentahydrate after pyrolysis at different temperatures. The inset shows the corresponding frequency-domain amplitude spectra obtained by Fourier transform of the time-domain signals, extending to higher frequencies where the signal intensity gradually decreases.

Terahertz radiation is exceedingly sensitive to changes in both the content and the state of water. As an initial approach, we employed a transmission-mode THz-TDS measurement scheme to characterize the dielectric response of CuSO_4_·5H_2_O at different thermal decomposition temperatures using THz-TDS. The terahertz time-domain spectra obtained at various temperatures are shown in Figure 1. The spectral amplitude increases with increasing temperature. The data also reveal a distinct time delay relative to the reference signal, accompanied by a notable attenuation. These observations indicate that the sample induces both absorption and a significant phase delay of the terahertz waves within this frequency range. The observed time delay of the transmitted pulse reflects the influence of the refractive index and its variation with temperature. In essence, the interaction of terahertz waves with the material arises from both absorption and refractive (phase) effects, which are influenced by water molecules and the evolving crystalline structures formed during thermal decomposition. The inset shows the corresponding frequency-domain amplitude spectra obtained by Fourier transform of the time-domain signals. Weak absorption features observed in the reference spectrum may be attributed to residual atmospheric water vapor, which is known to exhibit characteristic absorption in the terahertz region.

Notably, the waveform of the 150 °C sample is shifted leftward (shorter time delay) compared to the 30 °C sample. The temporal position of the THz waveform is influenced by both the sample thickness and its effective refractive index. Although minor variations in pellet thickness exist due to sample preparation, their relative deviation is limited and does not show a systematic trend with temperature. Therefore, the observed time shift is more likely associated with changes in the refractive index during thermal decomposition. In particular, the noticeable shift at 150 °C may be related to intermediate dehydration stages, where structural and compositional evolution affects the dielectric properties.

In copper(II) sulfate pentahydrate, water molecules exist in two primary forms: those directly coordinated to the Cu^2+^ ions and those engaged in hydrogen bonding. Both types play crucial roles in shaping the structural and chemical properties of the hydrate. The variations observed in the amplitude of the THz time-domain signals can be attributed mainly to two factors: the progressive loss of crystalline water during thermal decomposition, and the inherently heterogeneous crystal symmetry and coordination environment characteristic of the hydrated CuSO_4_·5H_2_O structure. These differences in dielectric behavior exert a significant influence on the optical response of terahertz radiation. The inset of Figure 1 presents the terahertz frequency-domain spectra obtained by Fourier transforming the reference and sample time-domain signals. As the temperature increases, both the spectral bandwidth and the spectral amplitude of the THz frequency-domain spectra exhibit notable changes. These variations arise from the temperature-dependent differences in the absorption capability of the sample toward terahertz waves.

According to independent thermal and structural characterization (e.g., TGA and XRD), CuSO_4_·5H_2_O undergoes progressive dehydration with increasing temperature, transitioning from pentahydrate to lower hydration states and eventually to anhydrous copper sulfate [10,29,30,34]. The corresponding variations observed in the THz absorbance spectra reflect the sensitivity of terahertz waves to changes in hydration state and dielectric properties. In addition, some spectra (e.g., 30 °C and 120 °C) exhibit distinct features, which may be associated with changes in hydration state and crystal structure during thermal decomposition. These variations can influence intermolecular interactions and lattice dynamics, leading to observable changes in THz absorption.

**Figure 2:**
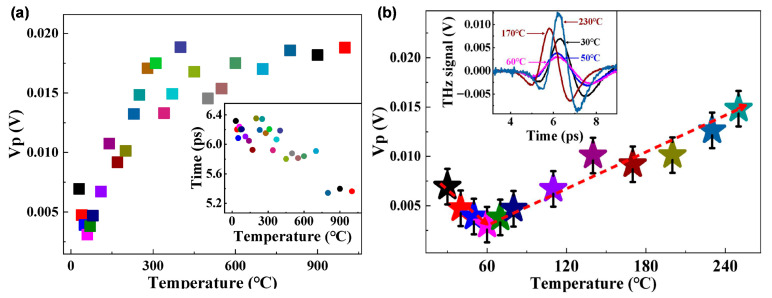
(**a**) Peak amplitude of the THz time-domain signal of copper sulfate pentahydrate at the temperature from 30 to 1000 °C, the inset shows the time delay of copper sulfate pentahydrate; (**b**) Peak amplitude of the THz time-domain signal of copper sulfate pentahydrate at the temperature from 30 to 250 °C, the inset shows the terahertz time domain spectroscopy of copper sulfate pentahydrate. Different colors represent samples treated at different temperatures. Error bars are included where applicable to indicate the variability of the measured data.

To investigate the dehydration process of copper(II) sulfate pentahydrate in greater detail, an extended temperature range was explored. Samples initially at ambient conditions were heated to temperatures from 30 °C up to 1000 °C, covering both dehydration and subsequent thermal decomposition stages. Figure 2a presents the peak amplitudes of the THz time-domain signals for samples treated over the full temperature range (30–1000 °C), with the inset showing the corresponding temporal delay. The time delay is used here as a direct observable reflecting phase evolution, enabling comparative analysis without requiring the extraction of absolute refractive index. The results indicate that the THz response continues to evolve even after the removal of crystalline water, reflecting ongoing structural and compositional changes at higher temperatures. A slight temporal shift in the THz waveform is observed with increasing temperature. As discussed, for Figure 1, this shift may be influenced by both sample thickness and the effective refractive index, and is more likely associated with temperature-induced variations in dielectric properties during thermal decomposition

To further highlight the temperature-dependent behavior during the dehydration stage, Figure 2b focuses on the lower temperature range (30–250 °C). The peak amplitude exhibits a non-monotonic trend, decreasing initially and reaching a minimum around 60 °C, followed by a gradual increase at higher temperatures. This behavior is consistent with the evolution of water content and intermediate hydration states during dehydration. The inset in Figure 2b provides additional insight into the temporal characteristics of the THz signals. Specifically, the full width at half maximum (FWHM) of the time-domain pulses gradually narrows as the dehydration process progresses. This change reflects a modification in the interaction between the THz wave and the material. The narrowing of the pulse width may be associated with reduced absorption and dispersion effects as water content decreases, leading to a less dispersive dielectric environment and a more compact temporal waveform. Therefore, the inset complements the amplitude-based analysis by revealing the evolution of pulse dynamics during thermal decomposition. The peak amplitude and time delay are used for relative comparison across samples rather than as absolute physical parameters.

During this pyrolysis stage, the products obtained at different temperatures are composed of copper sulfate molecules containing varying amounts of crystalline water. Since the THz absorption of the samples originates from both copper sulfate and water, the states of crystalline water become the primary factor responsible for the variations in the spectral responses. Moreover, water molecules in CuSO_4_·5H_2_O are highly sensitive to temperature and its fluctuations, leading to distinct THz absorption behaviors at different pyrolysis temperatures. The THz absorption first increases and then decreases with rising temperature. This behavior can be explained as follows: although some water molecules dissociate from the crystal lattice at temperatures below the boiling point of water (100 °C), they are unable to evaporate efficiently due to insufficient thermal energy. Meanwhile, anhydrous copper sulfate exhibits strong hygroscopicity. Therefore, at relatively low temperatures, both dehydration and rehydration processes occur simultaneously: lattice-bound water is thermally released, whereas nearby free water molecules are reabsorbed by copper sulfate through hydration and the proportion of free water in the sample becomes increasingly higher. When the temperature approaches 60 °C, a dynamic equilibrium is established between crystalline water within the lattice and free water outside the lattice, resulting in the highest proportion of free water in the sample [31,32,35]. Consequently, the THz absorption at 60 °C arises from combined contributions of bound water, free water, and copper sulfate molecules, leading to the maximum absorption intensity. As the temperature further increases, the previously non-evaporable water molecules gain sufficient energy to vaporize and escape into the environment, disrupting the earlier equilibrium. This results in a gradual decline in THz absorption above 60 °C. Overall, the results demonstrate that terahertz time-domain spectroscopy exhibits high sensitivity in detecting the content and state of water.

### 2.2. Quantitative Analysis of Refractive Index and Absorption Coefficient

To provide a more quantitative understanding of the terahertz response, the refractive index (*n*) and absorption coefficient (α) of CuSO_4_·5H_2_O at different thermal decomposition temperatures were calculated based on the THz-TDS measurements [36]. The refractive index was determined from the time delay (Δt) between the sample and reference signals, while the absorption coefficient was calculated from the amplitude attenuation. The formula for calculating the absorption coefficient [36] is:

(1) $$\alpha =-\frac{2}{d}\ln[\frac{{\left(n+1\right)}^{2}}{4n}\frac{E_{s}}{E_{r}}]$$

where *d* = 0.2 cm in the equation. Considering that the polyethylene-mixed pellets contain inherent porosity, a correction based on effective medium theory was further applied to obtain the intrinsic refractive index (*n*_intrinsic_) [36] and the formula is:

(2) $$n_{\mathrm{intrinsic}}=\frac{n-f}{1-f}$$

As the temperature increases, CuSO_4_·5H_2_O gradually loses water to form CuSO_4_·3H_2_O, CuSO_4_·H_2_O and CuSO_4_. For different hydration states, the corresponding porosity values were listed in Table 1.

The calculated refractive index and intrinsic refractive index are summarized in Table 2. Table 2 shows that the measured refractive index (*n*) ranges from approximately 1.541 to 1.622, while the intrinsic refractive index (*n*_intrinsic_) is slightly higher, ranging from 1.638 to 1.777 after porosity correction. This difference reflects the influence of air voids within the pellets, which effectively reduce the measured dielectric constant.

**Figure 3:**
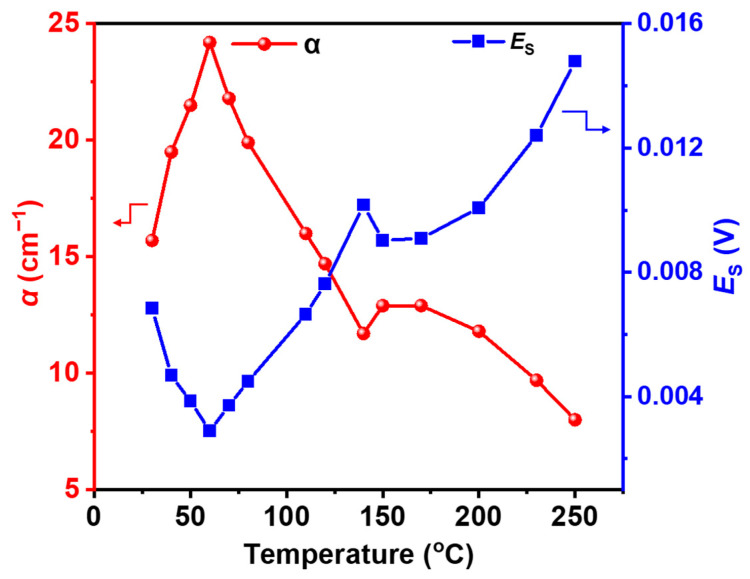
Absorption coefficient (α) and the transmitted electric field (*E*_S_) of polyethylene-mixed pellet at different temperatures. Note: Reference amplitude *E*_r_ = 0.03292 V.

The absorption coefficient (α), also summarized in Figure 3, shows a clear dependence on temperature. THz signal (V) is directly proportional to *E*(V). *E*_S_ represents the transmitted electric field amplitude used for calculating α. At lower temperatures (30–60 °C), α increases and reaches a maximum value (~24.2 cm^−1^ at 60 °C). As the temperature increases further, α gradually decreases.

This trend is consistent with the known dehydration pathway of CuSO_4_·5H_2_O, where the material transforms from pentahydrate to lower hydration states and eventually to anhydrous CuSO_4_. Overall, the introduction of refractive index and absorption coefficient provides a more physically meaningful interpretation of the terahertz response, complementing the qualitative analysis based on time-domain signals.

### 2.3. Terahertz-Response-Driven Analysis of the Pyrolysis Behavior of Crystalline Water

To further elucidate the working mechanism of THz-TDS, TGA was employed to determine the temperature ranges corresponding to the release of water molecules from CuSO_4_·5H_2_O. XRD was used to identify the crystalline phases of the pyrolysis products obtained at different temperatures, and SEM was applied to characterize their morphological evolution.

**Figure 4:**
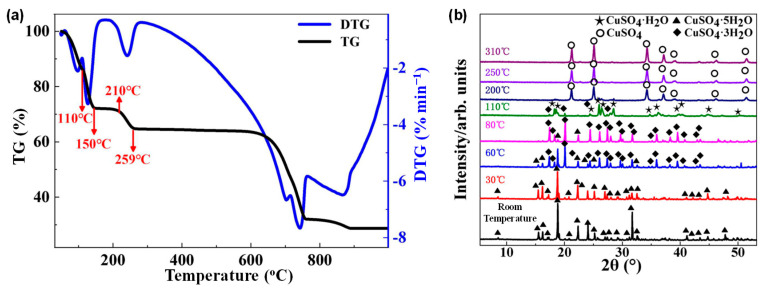
(**a**) Thermogravimetric and differential thermal analysis curves of copper sulfate pentahydrate; (**b**) The X-ray diffraction pattern of copper sulfate pentahydrate after pyrolysis at different temperatures.

Figure 4a presents the TGA curve of CuSO_4_·5H_2_O recorded at a heating rate of 10 °C min^−1^. A distinct multi-stage mass-loss process is observed during heating. Detailed examination of the TG and DTG curves reveals that the dehydration of CuSO_4_·5H_2_O proceeds through three stages, in which the dehydration temperatures of CuSO_4_·3H_2_O and CuSO_4_·H_2_O occur at approximately 110 °C and 210 °C, respectively. During the temperature range of 150–210 °C, a total of four water molecules are released, resulting in a mass loss of 27.82 wt%. These results indicate that TGA alone is insufficient for accurately determining the kinetic parameters of the dehydration process of CuSO_4_·5H_2_O. On one hand, the relatively small sample mass used in TGA experiments may lead to large relative errors. On the other hand, different heating conditions may induce structural variations in the dehydrated products. THz-TDS can thus serve as a complementary technique to TGA: (i) THz measurements typically involve larger sample quantities, which reduces relative error; and (ii) THz-TDS is highly sensitive to structural changes within the material during dehydration.

Given that the pyrolysis products directly influence the terahertz response and the molecular structure of CuSO_4_·5H_2_O evolves continuously with temperature, XRD was performed to determine the phase composition of the samples obtained at different thermal decomposition temperatures. Figure 4b shows the XRD patterns of CuSO_4_·5H_2_O subjected to various heating temperatures. A clear temperature-dependent phase evolution is observed. At 30 °C, the diffraction peaks correspond predominantly to CuSO_4_·5H_2_O. When the temperature increases to 60 °C, slight shifts in peak positions and intensities occur, indicating partial transformation of CuSO_4_·5H_2_O into CuSO_4_·3H_2_O, as evidenced by the disappearance of some pentahydrate peaks and the emergence of new peaks associated with the trihydrate. Upon further heating to 80 °C, the characteristic peaks of CuSO_4_·3H_2_O become more pronounced. When the temperature reaches 110 °C, the material undergoes continuous dehydration, and the diffraction pattern is dominated by CuSO_4_·3H_2_O and CuSO_4_·H_2_O. At 200 °C, the sample is almost entirely converted to anhydrous CuSO_4_.

These XRD results confirm that the dehydration of CuSO_4_·5H_2_O proceeds sequentially through CuSO_4_·5H_2_O → CuSO_4_·3H_2_O → CuSO_4_·H_2_O → CuSO_4_, accompanied by continuous structural rearrangements during pyrolysis. Since the crystalline water in CuSO_4_·5H_2_O is highly sensitive to temperature, the mass-loss percentages at each stage provide an accurate representation of its dehydration pathway.

The results indicate that CuSO_4_·5H_2_O first loses two water molecules during the initial stage of pyrolysis, followed by the release of another two water molecules as the decomposition proceeds. After a subsequent relatively stable stage, the final water molecule is removed. Because the dehydration rate of CuSO_4_·5H_2_O is influenced by both the heating rate and environmental conditions, the mass loss below 110 °C appears as a continuous process. Nevertheless, the specific dehydration steps can be clearly identified from the DTG curve.

By correlating the THz-TDS results (Figure 2) with the thermogravimetric and XRD analyses (Figure 4), a consistent interpretation of the dehydration process can be established. The variations observed in the THz peak amplitude and absorbance correspond to distinct stages of water loss and structural transformation. In particular, the minimum in peak amplitude around 60 °C coincides with the initial dehydration stage, as supported by TG and XRD results. The subsequent increase in peak amplitude and gradual evolution of absorbance correspond to further dehydration between 110 °C and 210 °C. At higher temperatures (e.g., around 400 °C), additional changes observed in the THz response are associated with post-dehydration structural transformations. Notably, the apparent difference between the step-like variations in the time-domain signals and the smoother trends in the frequency-domain spectra arises from the different physical quantities they represent, yet both consistently reflect the intrinsic thermal evolution of the material.

**Figure 5:**
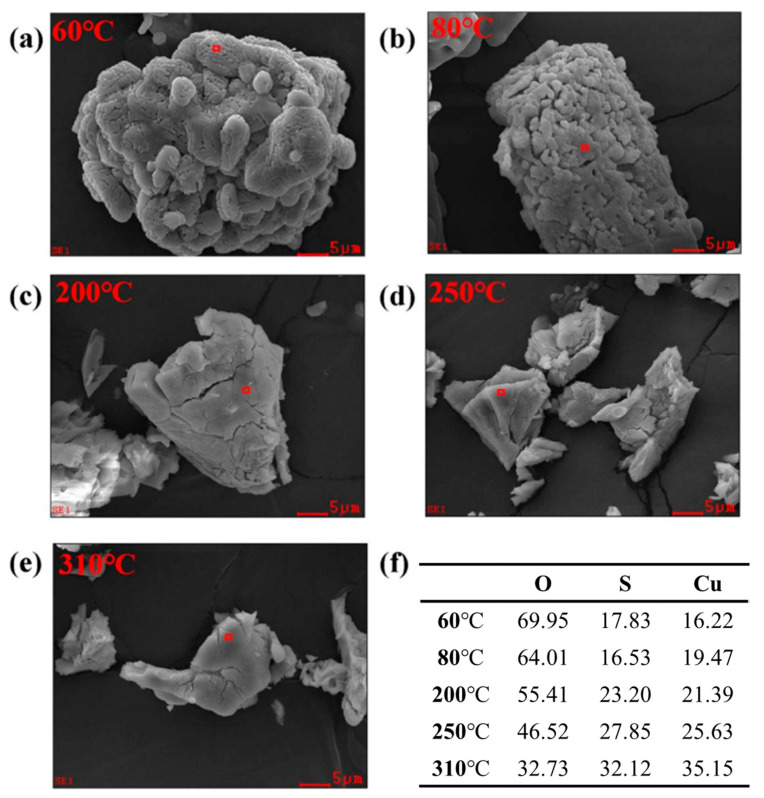
SEM and EDS analysis of copper sulfate pentahydrate at different pyrolysis temperatures. (**a**–**e**) SEM images of pyrolyzed copper sulfate pentahydrate at 60 °C, 80 °C, 200 °C, 250 °C and 310 °C, respectively. Red boxes mark the areas selected for EDS analysis. (**f**) Elemental composition and content of pyrolyzed copper sulfate pentahydrate.

SEM and EDS are essential tools for evaluating the relationship between microstructural features and physicochemical properties. SEM images of CuSO_4_·5H_2_O subjected to different pyrolysis temperatures are shown in Figure 5. The results indicate that the significant differences in water content among the samples lead to distinct variations in porosity. This is because pure anhydrous CuSO_4_ exists as a powder and exhibits strong hygroscopicity at relatively low temperatures; thus, the incorporation of crystalline water transforms the material from a powdery to a crystalline state. During the thermal dehydration of CuSO_4_·5H_2_O, the removal of coordinated water molecules generates pores and micro-cracks within the sample. Further analysis of the corresponding EDS data confirms that variations in the number of water molecules are reflected in changes in the elemental composition, ratios, and relative contents of the copper sulfate species.

By integrating the thermogravimetric (TG) data, SEM observations, and EDS results, the states and quantities of water molecules within CuSO_4_·5H_2_O at each pyrolysis stage can be reasonably evaluated. These complementary datasets also provide strong validation for the trends observed in the THz-TDS measurements. The combined evidence demonstrates that THz-TDS is capable of effectively characterizing both the forms and contents of water in mineral systems. Moreover, THz-TDS serves as an effective supplement to conventional analytical techniques, enhancing the diversity of characterization methods while improving the reliability and scientific robustness of the analytical outcomes.

## 3. Materials and Methods

### 3.1. Materials

CuSO_4_·5H_2_O with a chemical purity higher than 99.0% was selected as a chemically pure and structurally well-defined hydrated compound. It was used as the model system to investigate the relationship between dehydration behavior and terahertz spectral response.

To ensure uniform heating behavior and reduce mechanical effects during sample preparation, the particle size of CuSO_4_·5H_2_O was controlled within the range of 40–60 μm using a standard sieve. This procedure minimized potential crystalline water loss caused by friction-induced heating during grinding and avoided changes in the heating behavior due to particle size heterogeneity.

Experiments at each thermal treatment temperature were performed in triplicate using three independent batches of CuSO_4_·5H_2_O. For each batch, three pellets were prepared and measured as described below. The reported THz parameters are the mean ± standard deviation of these nine measurements.

### 3.2. Thermal Treatment Procedure

**Figure 6:**
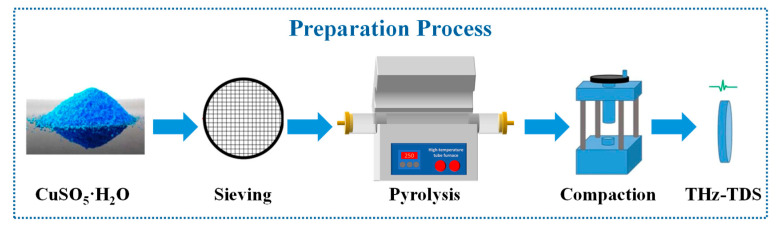
Preparation procedure of the CuSO_4_·5H_2_O sample.

The thermal decomposition of CuSO_4_·5H_2_O was carried out in a tubular furnace under controlled heating conditions. The particles were heated separately to the following temperatures: 40 °C, 50 °C, 60 °C, 70 °C, 80 °C, 110 °C, 140 °C, 170 °C, 200 °C, 230 °C, and 250 °C. These temperature points were selected to cover the stepwise dehydration process of copper sulfate pentahydrate, including the transition from pentahydrate to lower hydration states and eventually to anhydrous CuSO_4_. After reaching the target temperature, samples were cooled to room temperature prior to terahertz characterization. The experimental workflow of the study is illustrated in Figure 6.

### 3.3. Terahertz Time-Domain Spectroscopy Measurements

**Figure 7:**
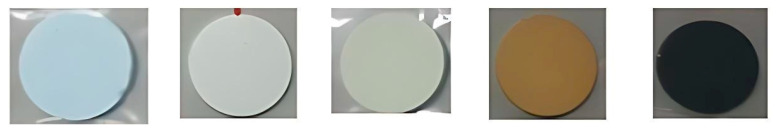
Copper sulfate pentahydrate samples at different pyrolysis temperatures.

THz-TDS was employed to investigate the thermal decomposition process of CuSO_4_·5H_2_O, with particular emphasis on how water content and the different states of water influence the terahertz spectral characteristics. For THz measurements, the thermally treated CuSO_4_·5H_2_O powder was mixed with high-density polyethylene (PE) powder at a volume ratio of 0.217:0.783 (CuSO_4_·5H_2_O:PE) to reduce scattering effects and improve terahertz transmission, rather than to modify the intrinsic porosity of the sample. The corresponding mass fraction of CuSO_4_·5H_2_O to HDPE was approximately 40:60. The total mass of each mixture was controlled at 1.6 g. The mixture was thoroughly homogenized by manual stirring for 5 min, then placed into a pellet die and rotated 100 times to ensure uniform distribution. Finally, the mixture was compressed under a pressure of 20 MPa for 2 min to form pellets with a diameter of 30 mm and a thickness of approximately 2 mm (measured using a micrometer caliper at multiple points), as shown in Figure 7.

**Figure 8:**
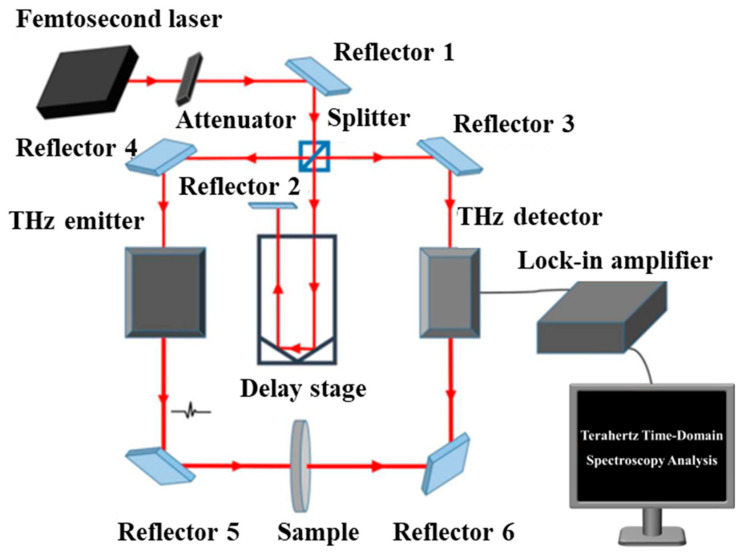
Schematic illustration of the THz-TDS experimental setup. Reflective elements are denoted as “Reflector” for general representation and do not imply a specific optical geometry.

THz measurements were carried out in transmission mode using a standard THz-TDS system (Figure 8). To minimize interference from atmospheric water vapor, a continuous flow of dry nitrogen was introduced into the measurement chamber during the experiments. The effective spectral range and resolution of the system were determined by the time-domain acquisition window and instrumental response, which limit the reliable frequency range for quantitative analysis.

The acquired time-domain signals were converted into frequency-domain spectra via fast Fourier transform (FFT). The spectral response was analyzed to evaluate the evolution of the material during thermal treatment, with particular attention to changes associated with water content and structural transformations.

In this work, both refractive index (*n*) and absorption coefficient (*α*) were extracted from the THz-TDS measurements based on the time-domain signals. Considering the presence of porosity in the polyethylene-mixed pellets, a porosity correction was further applied to obtain the intrinsic refractive index (*n*_intrinsic_), following established effective medium models, to provide quantitative evaluation of the dielectric response.

### 3.4. THz-TDS Measurement Conditions

All THz measurements were performed in a transmission-mode terahertz time-domain spectroscopy system. The measurement chamber was purged with dry nitrogen to maintain a relative humidity below 5%, and the ambient temperature was kept at 21 ± 1 °C. To minimize the influence of system drift, an improved acquisition method was adopted: the reference signal (without sample) and the sample signal were recorded in the same scan by rapidly inserting the sample pellet after the reference waveform was acquired. Each pellet was measured at three different points, and the averaged time-domain waveform was used for subsequent analysis. The time-domain signals were transformed into frequency-domain spectra via fast Fourier transform (FFT).

### 3.5. Thermogravimetric Analysis (TGA)

TGA was performed using a Mettler Toledo TGA 2 instrument (Mettler Toledo, Greifensee, Switzerland). A suitable amount (approximately 10 mg) of CuSO_4_·5H_2_O powder was placed in a crucible and heated from room temperature to 1000 °C at a heating rate of 10 °C/min under a nitrogen atmosphere (flow rate 50 mL/min). The mass variation as a function of temperature was continuously recorded. The stepwise weight-loss regions observed in the TGA curves were used to determine the temperature intervals corresponding to different hydration states. These results served as a reference for selecting the thermal treatment temperatures applied in the terahertz experiments and for correlating spectral evolution with structural dehydration stages.

### 3.6. X-Ray Diffraction (XRD)

XRD measurements were carried out on a Bruker D8 Advance diffractometer (Bruker AXS GmbH, Karlsruhe, Germany) (Cu Kα radiation, λ = 1.5406 Å, 40 kV, 40 mA) to investigate the phase evolution of CuSO_4_·5H_2_O during thermal treatment. Powder samples heated to different temperatures were subjected to XRD analysis to identify structural transformations from the pentahydrate to intermediate hydrates and finally to anhydrous CuSO_4_. At each temperature point, diffraction data were acquired in Bragg–Brentano geometry with a step size of 0.02° and a scan rate of 10° min^−1^ over the 2θ range of 5° to 55°. The diffraction peaks were obtained compared to standard reference data to determine phase composition. Changes in characteristic diffraction peaks were analyzed to monitor the gradual removal of crystalline water and the corresponding structural rearrangements.

### 3.7. Scanning Electron Microscopy (SEM)

SEM (JEOL JSM-7800, JEOL Ltd., Tokyo, Japan) was employed to examine the morphological evolution of CuSO_4_·5H_2_O particles during thermal dehydration. Powder samples corresponding to different dehydration stages (60 °C, 80 °C, 200 °C, 250 °C, and 310 °C) were mounted for SEM observation. The surface morphology and microstructural features were analyzed to evaluate potential structural collapse, particle fragmentation, or surface evolution induced by the progressive loss of crystalline water. Energy-dispersive X-ray spectroscopy (EDS) was performed simultaneously to determine the elemental composition and verify the stability of Cu and S elements throughout the thermal process. The elemental distribution was analyzed to confirm that the observed mass loss was primarily attributed to the removal of water molecules rather than compositional decomposition of the sulfate framework. The combined SEM and EDS results provided complementary morphological and compositional evidence supporting the dehydration mechanism inferred from THz and TGA analyses.

## 4. Conclusions

In this study, CuSO_4_·5H_2_O, a chemically pure hydrated mineral, was selected to investigate how the content and state of water influence terahertz spectral responses. The experimental results confirm that the dehydration of CuSO_4_·5H_2_O occurs in a stepwise manner, proceeding sequentially through CuSO_4_·5H_2_O, CuSO_4_·3H_2_O, CuSO_4_·H_2_O, and finally anhydrous CuSO_4_. Beyond this stepwise mass loss, the THz-TDS measurements reveal an additional phenomenon: water molecules released from the crystal lattice do not immediately evaporate but remain temporarily attached to the surrounding hydrated copper sulfate in the form of free water. A dynamic equilibrium between free water and coordinated water is established at approximately 60 °C, corresponding to the strongest terahertz absorption observed among all samples.

Overall, the results demonstrate that terahertz time-domain spectroscopy is highly sensitive to subtle variations in water content and state, indicating that even minor dehydration processes can be effectively monitored. The abrupt changes in terahertz parameters during heating suggest that THz-TDS can serve as a powerful tool for probing the evolution of water states throughout the thermal decomposition of hydrated minerals. Furthermore, the THz-derived findings were independently verified through TGA, XRD, and SEM, confirming both the accuracy and reliability of the terahertz-based approach. Therefore, THz-TDS represents an effective and promising technique for determining the presence, quantity, and bonding states of water in mineral systems, offering valuable complementary insights alongside traditional analytical methods.

Future work could extend this approach to more complex mineral systems with multiple hydration states or to in situ monitoring of dehydration processes under controlled humidity and temperature conditions. The development of portable THz systems may eventually enable field applications in mineral exploration and resource assessment. Additionally, combining THz-TDS with computational modeling could provide deeper insights into the molecular dynamics of water release and the structural rearrangements accompanying dehydration.

## Figures and Tables

**Table 1:** The corresponding porosity values for different hydration states. Data errors (*f*) = 0.1%.

Material	HDPE Mixed with CuSO_4_∙5H_2_O	HDPE Mixed with CuSO_4_∙3H_2_O	HDPE Mixed with CuSO_4_∙H_2_O	HDPE Mixed with CuSO_4_
porosity (*f*)	8.7%	14.5%	18.4%	20.5%

**Table 2:** Time delay, refractive index (*n*), and intrinsic refractive index (*n*_intrinsic_) of polyethylene-mixed pellet at different temperatures. Data errors (*n*) = 0.001 and data errors (*n*_intrinsic_) = 0.001.

Temperature (°C)	30	50	60	80	120	150	170	230	250
Δt (ps)	4.09	3.89	3.95	4.00	4.15	3.88	3.61	4.07	4.12
*n*	1.612	1.583	1.592	1.599	1.622	1.582	1.541	1.610	1.618
*n*_intrinsic_	1.671	1.638	1.648	1.701	1.763	1.713	1.663	1.767	1.777

## Data Availability

The original contributions presented in this study are included in the article. Further inquiries can be directed to the corresponding authors.
